# Supplementary figures and images for: Coordinating the morphogenesis-differentiation balance by tweaking the cytokinin-gibberellin equilibrium
Source: PLoS Genet. 2021 Apr 26;17(4):e1009537. doi: 10.1371/journal.pgen.1009537 (PMC8102002; doi:10.1371/journal.pgen.1009537)

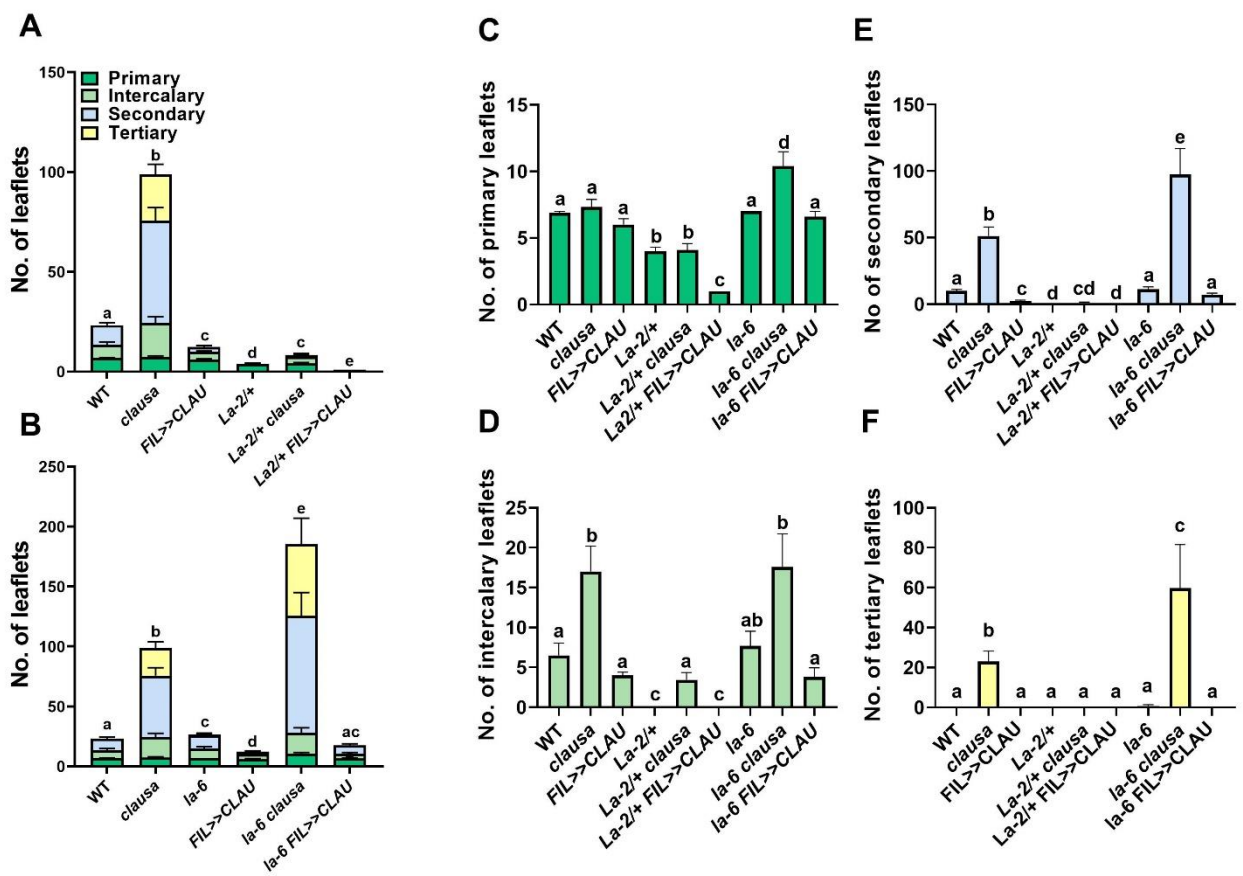

S1 Fig.

Supplement: S1 Fig — (A-F) Quantification of leaf complexity in genotypes with altered CLAU and LA expression levels. Graphs represent mean ± SE of six independent biological repeats. (A-B) Stacked bars of total leaflet number split into leaflet types, bars = SE. Statistical significance of differences in the total leaflet number was examined in a one-way ANOVA, p<0.0001. Different letters indicate significant differences between samples in an unpaired two-tailed t-test with Welch’s correction. (C-F) Graphs representing each leaflet type separately. Statistical significance of differences was examined in a one-way ANOVA, p<0.0001. Different letters indicate significant differences between samples in a Tukey post hoc test (C) or a two-tailed t-test (D-F), (C) p<0.0443, (D) p<0.0039, (E) p<0. 045, (F) p<0.022. (PDF) [file pgen.1009537.s001.pdf]

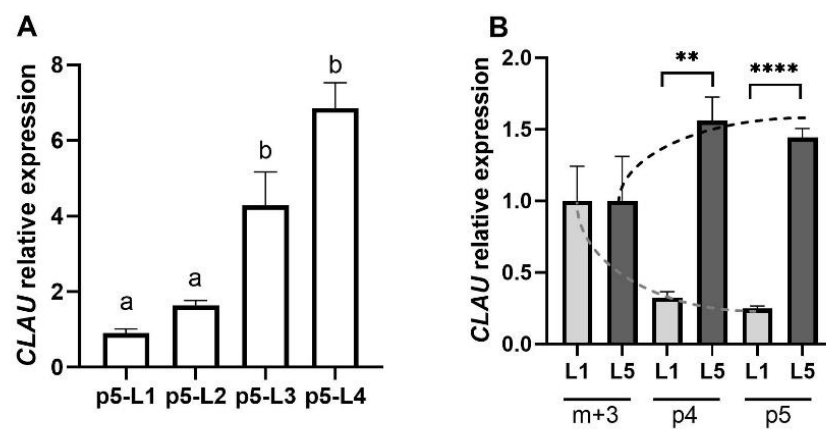

**S2 Fig.**

Supplement: S2 Fig — Expression level of CLAU was determined using RT-qPCR. (A) CLAU expression in the fifth plastochron in leaves 1 through 4. Leaves 1 and 2, which have reduced complexity, also have reduced CLAU expression. Graph represents mean ± SE of three independent biological repeats. Letters indicate significant differences in a one-way ANOVA with a Tukey post-hoc test, p≤0.037. (B) CLAU expression in successive leaf developmental stages, comparing the first and fifth leaves. Dashed line indicates expression trend. Graph represents mean ± SE of three independent biological repeats. Asterisks indicate significant differences in an unpaired, two-tailed t-test, p≤0.0019. (PDF) [file pgen.1009537.s002.pdf]

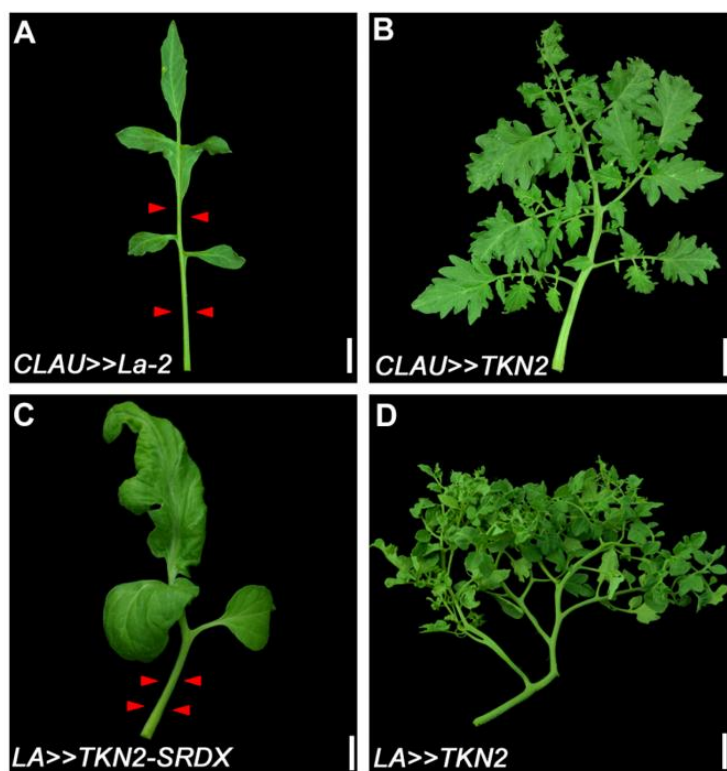

S3 Fig.

Supplement: S3 Fig — (A-D) Phenotypes of leaves of the indicated genotypes. All leaves depicted are fully expanded fifth leaves. Bars = 2 cm. (PDF) [file pgen.1009537.s003.pdf]

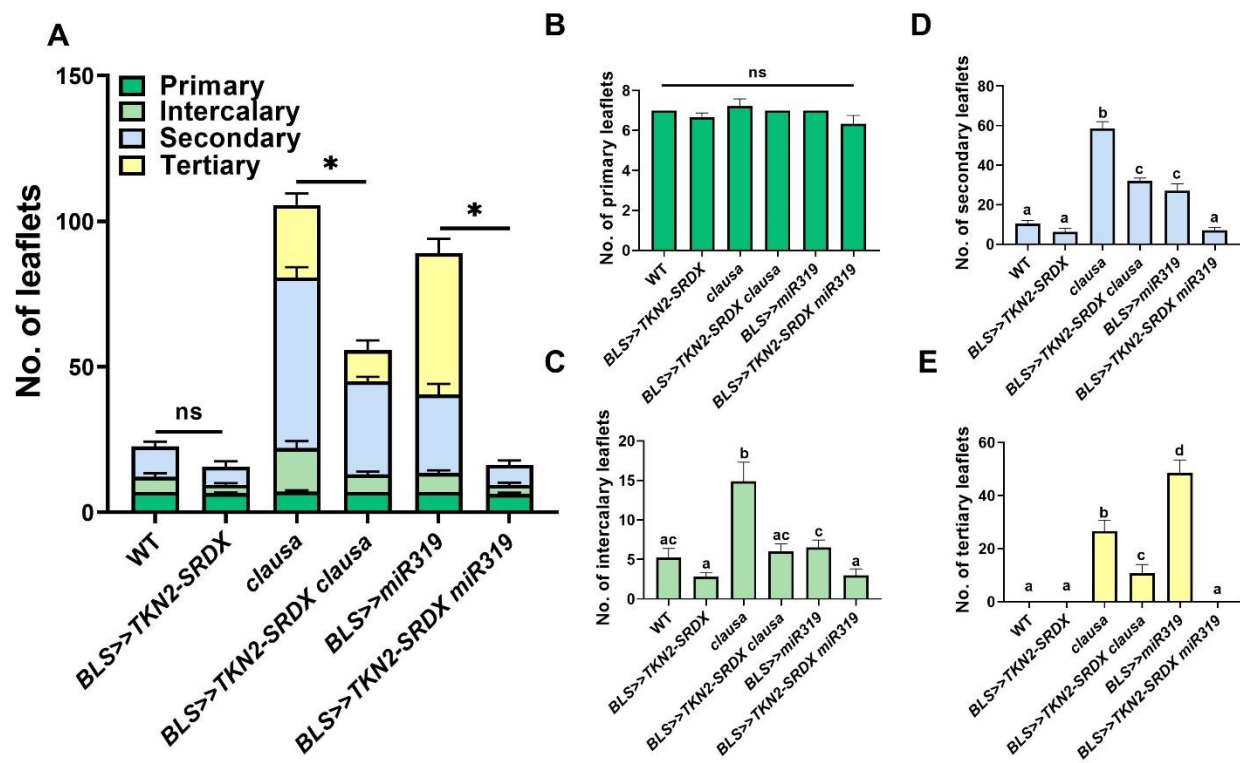

S4 Fig.

Supplement: S4 Fig — (A-E) Quantification of leaf complexity upon overexpression of TKN2-SRDX in the background of CLAU and LA deficiency. Graphs represent mean ± SE of at least three independent biological repeats. (A) Stacked bars of total leaflet number split into leaflet types, bars = SE. Asterisks indicate significant differences of the total leaflet number from the background genotype (without TKN2-SRDX overexpression) in an unpaired two-tailed t-test with Welch’s correction, p≤0.0335. (B-E) Graphs representing each leaflet type separately. Statistical significance of differences was examined in a one-way ANOVA, p<0.0001 (C-E). Different letters indicate significant differences between samples in a two-tailed t-test (B,C,E) or a Tukey post hoc test (D), (B) p = ns (no significant differences), (C) p<0.0249, (D) p<0.0052, (E) p<0.049. (PDF) [file pgen.1009537.s004.pdf]

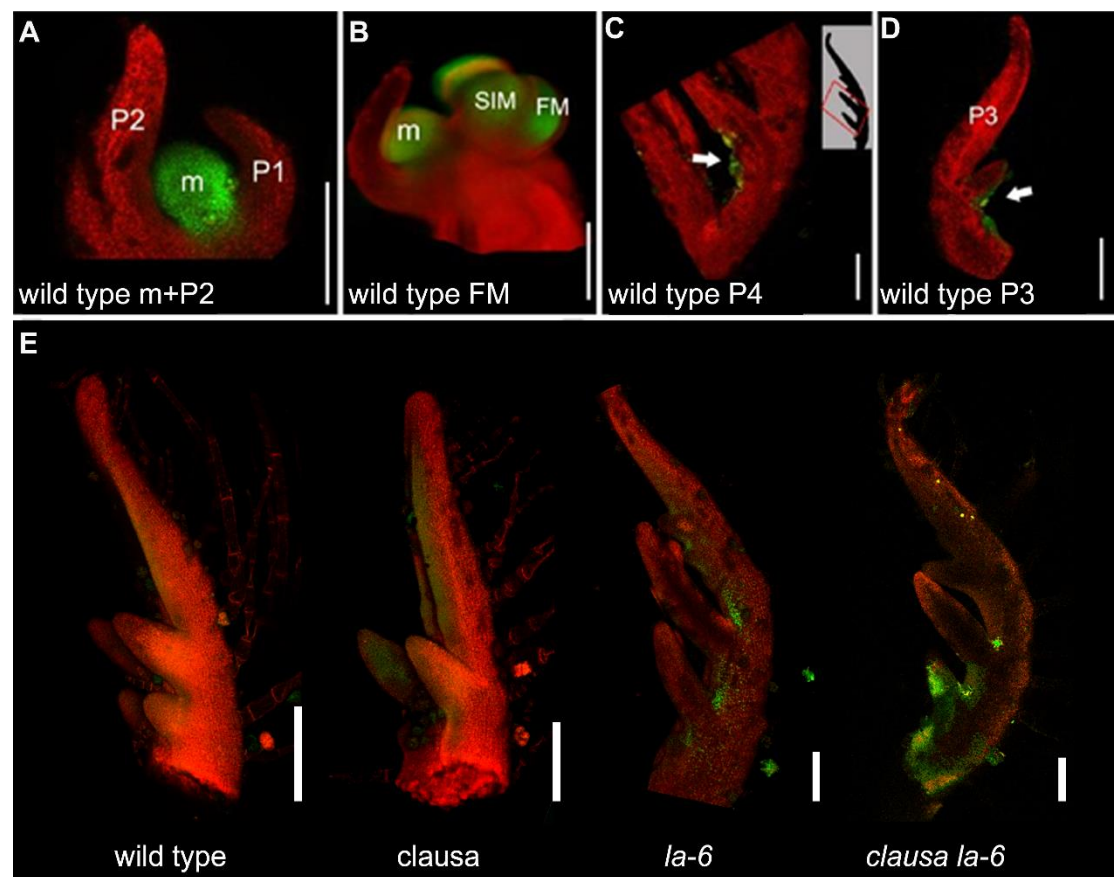

S5 Fig.

Supplement: S5 Fig — TKN2 is expressed at the leaf margin in CLAU and LA deficient backgrounds (A-D) Expression pattern of the TKN2 promoter fused to YFP in WT M82 plants. (A) Vegetative meristem (m) and two youngest leaf primordia (P1, P2). (B) Floral meristem (FM), sympodial inflorescence meristem (SIM). (C-D) Weak expression is observed in P3/P4 leaf primordia (area of the leaf primordium depicted in C is indicated in the inset). (E) Confocal micrographs of pTKN::nYFP in the fourth plastochron (P4) in indicated genotypes. TKN2 promoter activation increases in the leaf margin in CLAU and LA deficient backgrounds. The pattern of YFP expression was detected by a confocal laser scanning microscope (CLSM model SP8; Leica), with the solid-state laser set at 514 nm for excitation and 530 nm for emission. Chlorophyll expression was detected at 488nm excitation/ 700nm emission. Bars = 200 um. (PDF) [file pgen.1009537.s005.pdf]

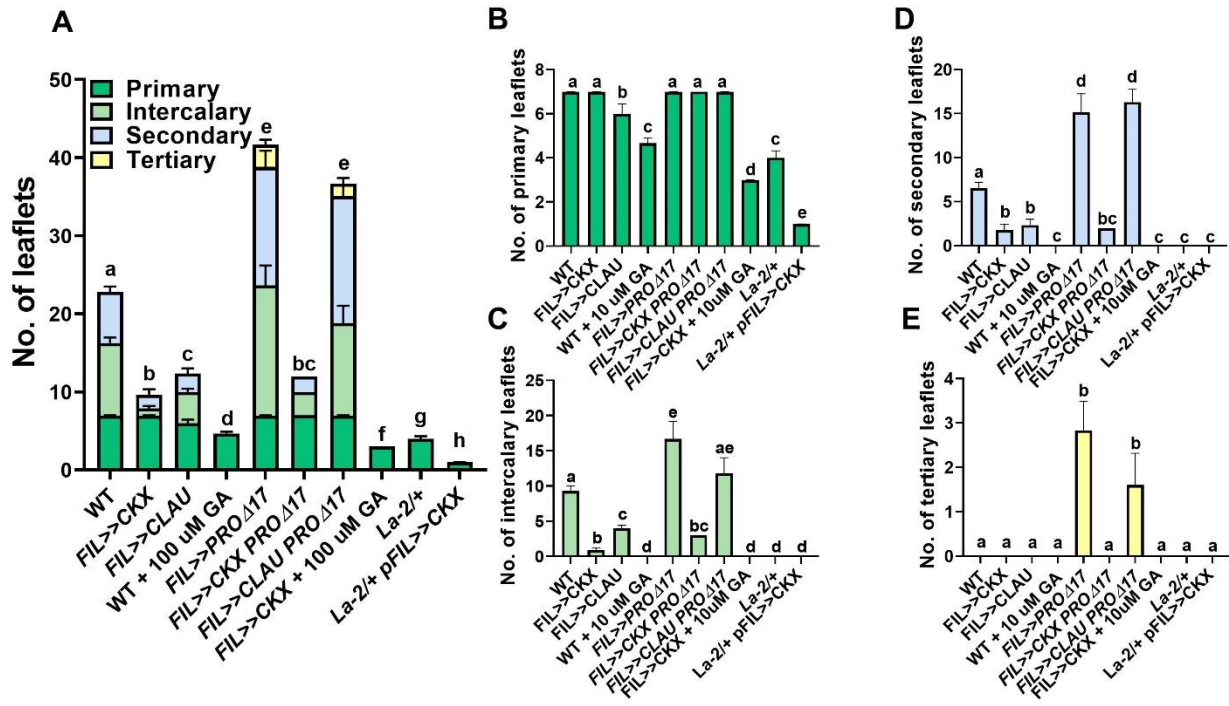

S6 Fig.

Supplement: S6 Fig — (A-E) Quantification of leaf complexity following GA treatment in WT and clausa. Graphs represent mean ± SE of at least three independent biological repeats. (A) stacked bars of total leaflet number split into leaflet types, bars = SE. Statistical significance of differences in the total leaflet number was examined in a one-way ANOVA, p<0.0001. Different letters indicate significant differences between samples in an unpaired two-tailed t-test with Welch’s correction (p<0.015). (B-E) Graphs representing each leaflet type separately. Statistical significance of differences was examined in a one-way ANOVA, p<0.0001. Different letters indicate significant differences between samples in a tukey post hoc test (B,E) or a two-tailed t-test (C,D), (B) p<0.0043, (C) p<0.011, (D) p<0.02, (E) p<0.03. (PDF) [file pgen.1009537.s006.pdf]

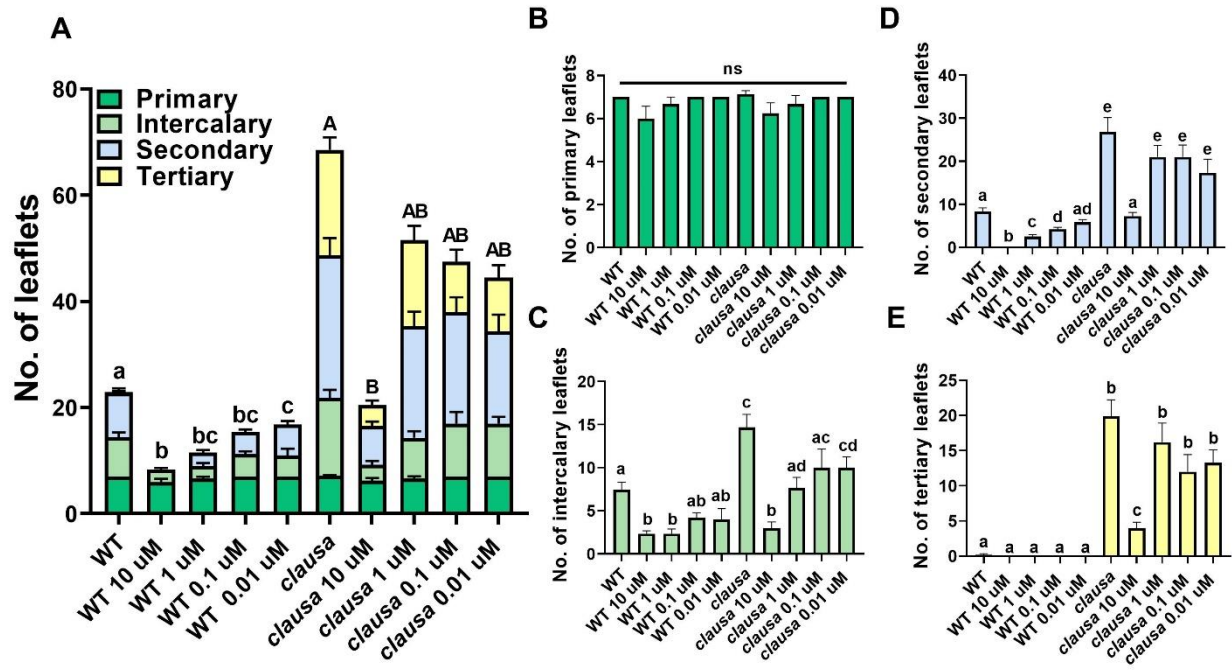

S7 Fig.

Supplement: S7 Fig — (A-E) Quantification of leaf complexity following GA treatment in WT and clausa. Graphs represent mean ± SE of at least three independent biological repeats. (A) stacked bars of total leaflet number split into leaflet types, bars = SE. (B-E) Graphs representing each leaflet type separately. Statistical significance of differences was examined in a one-way ANOVA, p<0.0001 (C-E). Different letters indicate significant differences between samples in a two-tailed t-test, (B) p = ns (no significant differences), (C) p<0.0255, (D) p<0.0398, (E) p<0.0212. (PDF) [file pgen.1009537.s007.pdf]

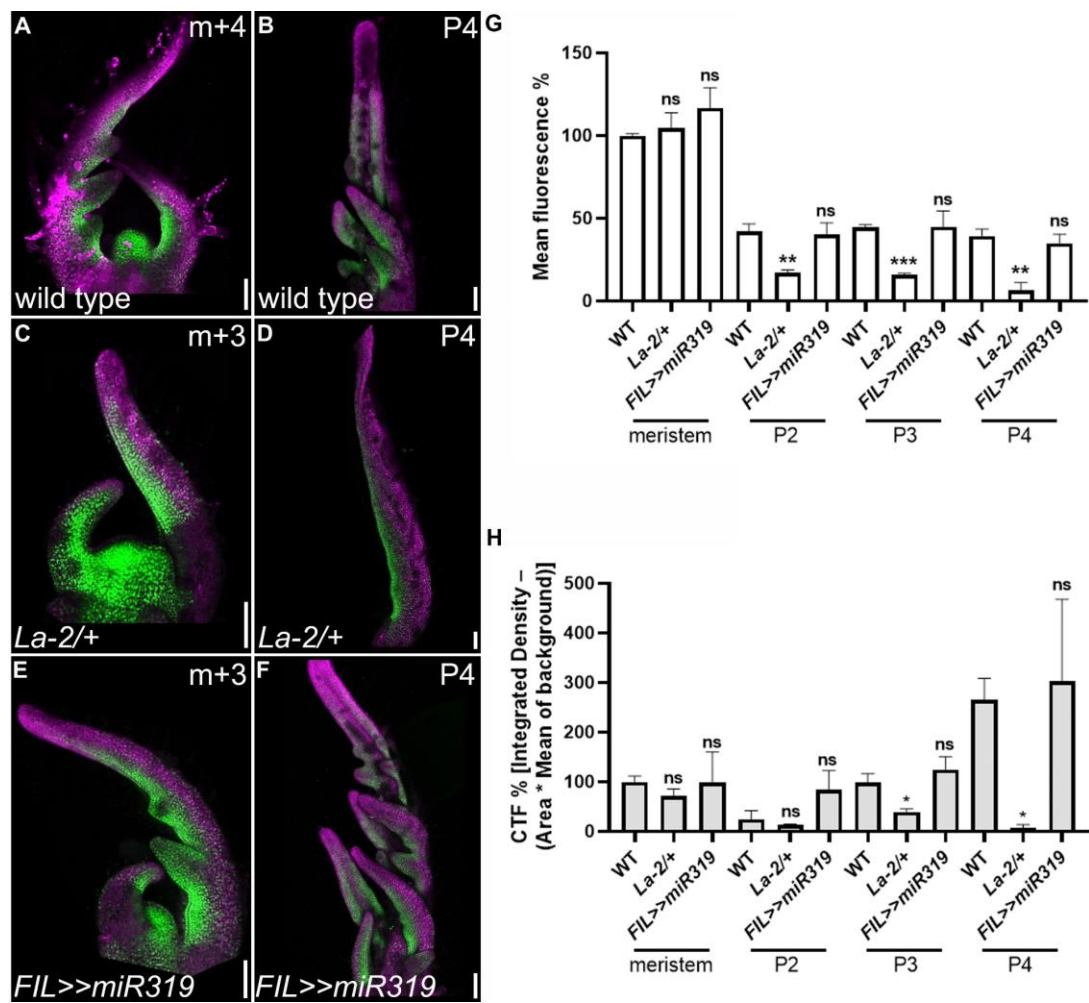

**S8 Fig.**

Supplement: S8 Fig — (A-F) Confocal micrographs of TCSv2::3XVENUS in successive developmental stages in indicated genotypes. TCSv2 driven signals are reduced in La-2/+ in the fifth plastochron. The pattern of VENUS expression was detected by a confocal laser scanning microscope (CLSM model SP8; Leica), with the solid-state laser set at 514 nm for excitation and 530 nm for emission. Chlorophyll emission was detected at 488nm excitation/ 700nm emission. Bars = 100 um. (G-H) Quantification of TCSv2 driven Venus fluorescence in indicated developmental stages of indicated genotypes. (J) Mean fluorescence % of WT meristem signal; (K) Corrected total fluorescence (CTF) was calculated as [integrated density–(area*background mean fluorescence)]- presented as % of WT meristem CTF. Quantifications were done on at least 5 plants from each genotype in 3 experiments. Asterisks indicate significance from WT of the same developmental stage in a two-tailed t-test, *p<0.05, **p<0.01, ***p<0.001, ns = not significant. (PDF) [file pgen.1009537.s008.pdf]

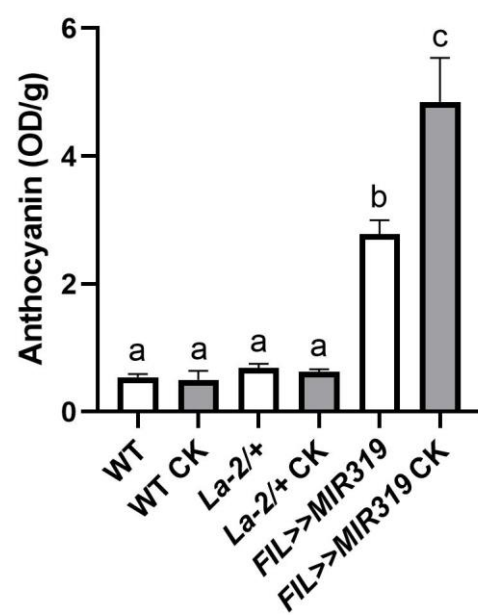

S9 Fig.

Supplement: S9 Fig — Anthocyanin content in WT and altered LA genotypes with or without CK treatment was determined by measuring optical density following methanolic extraction. Graph represents mean ± SE of five independent biological repeats. Letters indicate significant differences in an unpaired, two-tailed t-test with Welch’s correction, p≤0.038. (PDF) [file pgen.1009537.s009.pdf]

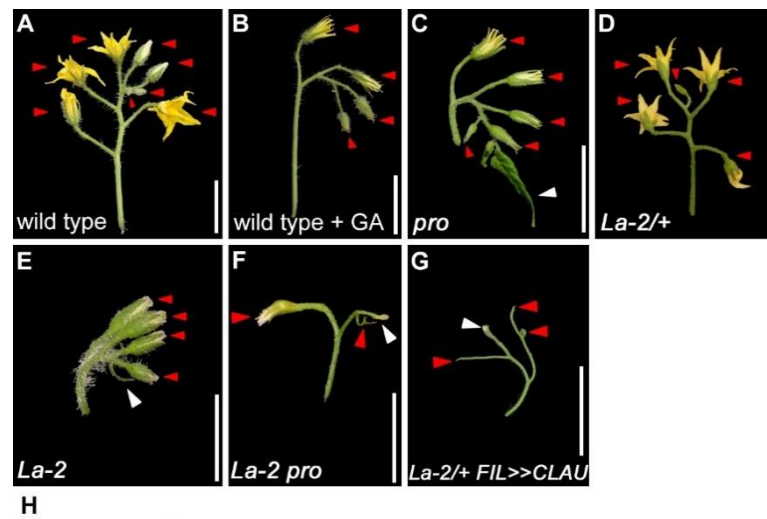

S11 Fig.

Supplement: S11 Fig — (A-H) Genetic interactions between genotypes with altered CLAU and LA expression levels and GA levels or response. La-2/+: a semi-dominant LA allele with increased and precocious expression due to miR319 resistance. FIL>>CLAU: CLAU upregulation. Increased GA levels (GA application) or response: in procera (pro) loss-of-function. Bars = 2 cm. Red and white arrowheads represent flowers and leaf-like structures, respectively. (I) Quantification of inflorescence complexity in the indicated genotypes and treatments. Graphs represent mean ± SE of at least 4 independent biological repeats. Letters indicate significant differences between samples in a one-way ANOVA with a Tukey post-hoc test, p<0.0009. (PDF) [file pgen.1009537.s011.pdf]
