# Supplementary material for: Coordinating the morphogenesis-differentiation balance by tweaking the cytokinin-gibberellin equilibrium
Source: PLoS Genet. 2021 Apr 26;17(4):e1009537. doi: 10.1371/journal.pgen.1009537 (PMC8102002; doi:10.1371/journal.pgen.1009537)
Supplement: S1 Table — (PDF) [file pgen.1009537.s012.pdf]

S1 Table.

Primers used in this work

**Name**	**5'-3' seq**	**Use**
pTKN2-R	CAC CCT GTC TCT AAT GAT CTC CAT CCC TA	pTKN2 cloning
pTKN2 -L	CAT TAT TCT CTC ACA CAC TTT CTT CTT	pTKN2 cloning
EXP RT-F	TGGGTGTGCCTTTCTGAATG	qRT-PCR
EXP RT-R	GCTAAGAACGCTGGACCTAATG	qRT-PCR
Clau F	CCTCTCACAACAAGCAATGAACTT	qRT-PCR
Clau R	AGGACGATGCAATGAGAGAGAC	qRT-PCR
GA20ox-1 F	AGATTGTGTTGGTGGACTTCAA	qRT-PCR
GA20ox-1 R	TAGCGCCATAAATGTGTCG	qRT-PCR
TRR16/17-1 F	AAGAATGGGAAGCAGCTAGATTTGGTCCCCATTATTAAGATTTAGCAAGA	EMSA probe generation
TRR16/17-1 R	TCTTGCTAAATCTTAATAATGGGGACCAAATCTAGCTGCTTCCCATTCTT	EMSA probe generation
TRR8/9-1 F	CTATTAGATTACACTTGTAAAACGTGTGGTCCAACATAGAGAAATGGTAATCTTTTTC	EMSA probe generation
TRR8/9-1 R	AAAAAGATTACCATTTCTCTATTGTTGGACCACACGTTTTACAAGTGTAATCTAATAGC	EMSA probe generation
TRR3/4-1 F	AAAAAAGGTAAATATATAAATTATGTAGGACCACAAAAAGATTTTGTAAAGATTAGACAA	EMSA probe generation
TRR3/4-1 R	TTGTCTAATCTTTACAAAATCTTTTTGTGGTCCTACATAATTTATATATTTACCTTTTTT	EMSA probe generation
TRR3/4-1m F	AAAAAAGGTAAATATATAAATTATGTAGGAACTCAAAAAGATTTTGTAAAGATTAGACAA	EMSA probe generation
TRR3/4-1m R	TTGTCTAATCTTTACAAAATCTTTTTGAGTTCCTACATAATTTATATATTTACCTTTTTT	EMSA probe generation
