# Supplementary material for: Coordinating the morphogenesis-differentiation balance by tweaking the cytokinin-gibberellin equilibrium
Source: PLoS Genet. 2021 Apr 26;17(4):e1009537. doi: 10.1371/journal.pgen.1009537 (PMC8102002; doi:10.1371/journal.pgen.1009537)
Supplement: S10 Fig — (A) LA binds in vitro to the promoters of TRRs. Recombinant MBP-LA caused a shift in the PAGE migration of biotinylated promoter derived probes (Probe-B) of the indicated TRRs. (B) Specific binding of LA to the TRR3/4 promoter. Recombinant MBP-LA caused a shift (s) in the PAGE migration of the biotinylated promoter derived probe (p) of TRR3/4. A non-biotinylated probe was able to compete with the biotinylated probe in a quantity depended manner (c1-c3). MBP (MBP empty) was used as a control protein and does not cause a probe shift in PAGE. (C) A biotinylated probe with a mutation in the binding site (m) does not shift in the presence of MBP-LA, and cannot compete with the WT probe (s+m1-m3). (PDF) [file pgen.1009537.s010.pdf]

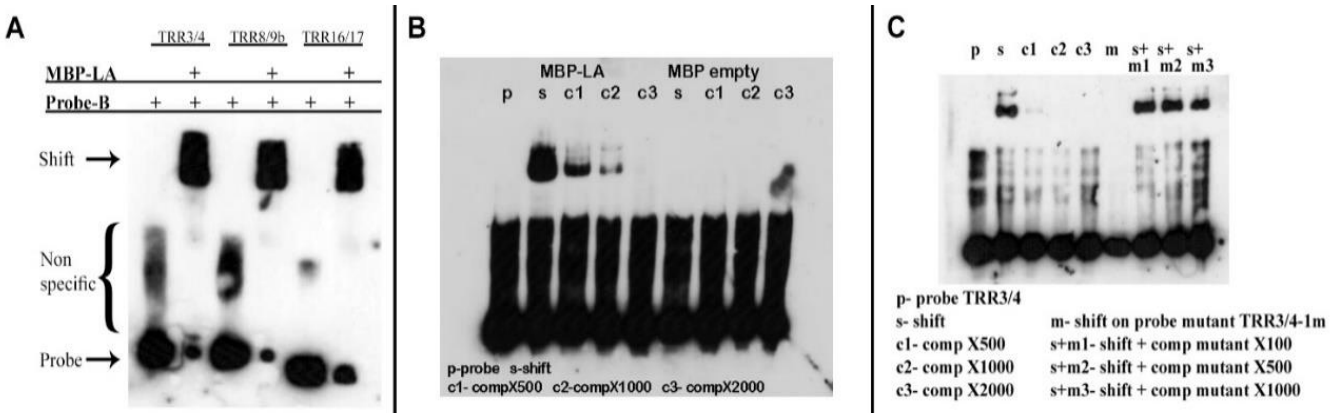
A
TRR3/4
TRR8/9b
TRR16/17
MBP-LA
Probe-B
Shift
Non specific
Probe
B
MBP-LA
MBP empty
p s c1 c2 c3 s c1 c2 c3
p-probe s-shift
c1- compX500 c2-compX1000 c3- compX2000
C
p s c1 c2 c3 m s+ m1 s+ m2 s+ m3
p- probe TRR3/4
s- shift
c1- comp X500
c2- comp X1000
c3- comp X2000
m- shift on probe mutant TRR3/4-1m
s+m1- shift + comp mutant X100
s+m2- shift + comp mutant X500
s+m3- shift + comp mutant X1000

S10 Fig.
