# Supplementary material for: Coordinating the morphogenesis-differentiation balance by tweaking the cytokinin-gibberellin equilibrium
Source: PLoS Genet. 2021 Apr 26;17(4):e1009537. doi: 10.1371/journal.pgen.1009537 (PMC8102002; doi:10.1371/journal.pgen.1009537)
Supplement: S4 Data — (PDF) [file pgen.1009537.s016.pdf]

Morphogenesis and differentiation genes were identified from a published dataset (Ichihashi *et al.*, 2014). The full lists are included in Supplemental dataset 3.

1824 genes fit the condition of m+P3 exp> P4 exp> P5 exp> P6 exp and are considered "Morphogenetic" genes.

1451 genes fit the condition of m+P3 exp< P4 exp< P5 exp< P6 exp and are considered "differentiative" genes.

"Morphogenetic" genes, as a group, are significantly enriched in the upregulated group in *clausa*, *pFIL>>miR319*, and *pFIL>>TKN2*, and in the downregulated group in *La2/+* and *pFIL>>TKN2-SRDX*. They are also significantly depleted in the downregulated group in *clausa* and *pFIL>>TKN2*, and in the upregulated group in *La2/+.*

**Genotype**	**Representation factor of "morphogenetic" genes**
clausa up	**Significantly over-represented**<br>**Representation factor: 1.5**<br>**p < 0.014**
clausa down	**Significantly under-represented**<br>**Representation factor: 0.4**<br>**p < 0.008**
La2/+ up	**Significantly under-represented**<br>**Representation factor: 0.6**<br>**p < 0.016**
La2/+ down	**Significantly over-represented**<br>**Representation factor: 1.4**<br>**p < 0.011**
pFIL>>miR319 up	**Significantly over-represented**<br>**Representation factor: 1.3**<br>**p < 0.044**
pFIL>>miR319 down	**Not significant**<br>**Representation factor: 1.2**<br>**p < 0.205**
pFIL>>TKN2 up	**Significantly over-represented**<br>**Representation factor: 2.2**<br>**p < 3.954e-28**
pFIL>>TKN2 down	**Significantly under-represented**<br>**Representation factor: 0.3**<br>**p < 1.710e-16**
pFIL>>TKN2-SRDX up	**Not significant**<br>**Representation factor: 1.1**<br>**p < 0.251**
pFIL>>TKN2-SRDX down	**Significantly over-represented**<br>**Representation factor: 1.6**<br>**p < 9.486e-06**

Interestingly, we see that the genes upregulated in *clausa* and *pFIL>>miR319* are different genes, supporting the notion that CLAU and LA operate in parallel pathways.

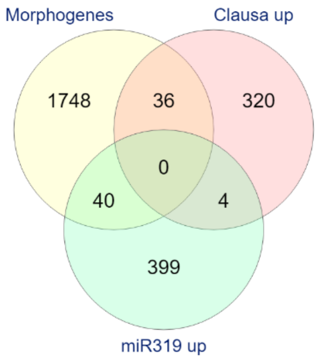

"Differentiative" genes, as a group, are significantly enriched in the upregulated group in *La2/+*, and in the downregulated group in *pFIL>>TKN2*, *clausa* and *pFIL>>miR319*. They are also significantly depleted in the downregulated group in La2/+ and in the upregulated group in *pFIL>>miR319*, *pFIL>>TKN2*, and, interestingly, *pFIL>>TKN2-SRDX*.

**Genotype**	**Representation factor of "differentiative" genes**
clausa up	**Not significant**<br>**Representation factor: 1.4**<br>**$p < 0.057$**
clausa down	**Significantly over-represented**<br>**Representation factor: 2.5**<br>**$p < 1.657e-06$**
La2/+ up	**Significantly over-represented**<br>**Representation factor: 4.0**<br>**$p < 2.309e-24$**
La2/+ down	**Significantly under-represented**<br>**Representation factor: 0.3**<br>**$p < 1.772e-04$**
pFIL>>miR319 up	**Significantly under-represented**<br>**Representation factor: 0.7**<br>**$p < 0.049$**
pFIL>>miR319 down	**Significantly over-represented**<br>**Representation factor: 2.6**<br>**$p < 2.808e-08$**
pFIL>>TKN2 up	**Significantly under-represented**<br>**Representation factor: 0.4**<br>**$p < 1.772e-09$**
pFIL>>TKN2 down	**Significantly over-represented**<br>**Representation factor: 2.7**<br>**$p < 9.404e-33$**
pFIL>>TKN2-SRDX up	**Significantly under-represented**<br>**Representation factor: 0.7**<br>**$p < 0.027$**
pFIL>>TKN2-SRDX down	**Not significant**<br>**Representation factor: 1.2**<br>**$p < 0.150$**

Once again, looking at the specific differentiation genes downregulated in *clausa* and *pFIL>>miR319*, we see very little overlap.

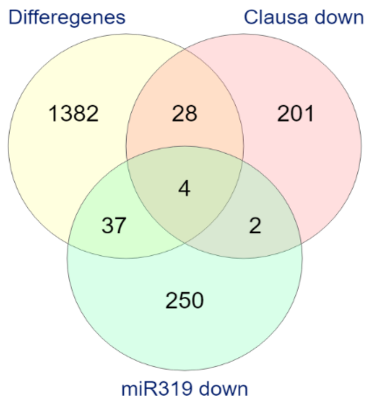

Ichihashi Y, Aguilar-Martinez JA, Farhi M, Chitwood DH, Kumar R, Millon L V, Peng J, Maloof JN, Sinha NR. **2014**. Evolutionary developmental transcriptomics reveals a gene network module regulating interspecific diversity in plant leaf shape. *Proc Natl Acad Sci U S A* **111**: E2616-21.
