# Supplementary material for: Coordinating the morphogenesis-differentiation balance by tweaking the cytokinin-gibberellin equilibrium
Source: PLoS Genet. 2021 Apr 26;17(4):e1009537. doi: 10.1371/journal.pgen.1009537 (PMC8102002; doi:10.1371/journal.pgen.1009537)
Supplement: Attachment — Submitted filename: 17-3-21 Decision letter - point by point .docx [file pgen.1009537.s017.docx]

Editor's comments:

One reviewer was enthusiastic and not very critical, the other reviewer was much more critical, but the criticism can probably be addressed in a resubmission.

Many thanks to all involved in the review process for their time and effort. We appreciate the positive response and the opportunity to submit a revised version of our manuscript.

Please find a point-by-point response to the editors and reviewers. All comments raised were addressed in the revised version of the manuscript. Where line numbers are indicated, they refer to the marked up version of the revised manuscript.

For example, the temporal action is mentioned but not demonstrated. Can you provide more information, (perhaps a cartoon) of the timing of expression for the different promoters?

We added a cartoon depicting the spatio-temporal expression of the different promoters used in this study to the revised version of Figures 1 and 3.

This reviewer also is concerned about the fact you treat all leaflets the same and just count them. Is it possible to address that criticism?

Many thanks for this comment. Indeed, we had this data for some of the experiments, and performed a new comprehensive phenotypic analysis, in which we quantified all the leaves in the different experiments at the different leaflet orders. The leaflet order quantification has been added to Figures 1, 3 and 5, with comprehensive statistical analyses of all leaflet orders in new supplemental figures S1, S4, S6 and S7. Leaflet order quantification is not provided for Figure 4, as most of those genotypes are extremely compound, making order identification and quantification difficult and unreliable. This is also why, for these genotypes, only lateral leaflet #2 was analyzed. This comment was very helpful and the resulting analysis strengthened some of our conclusions. In general, it clarifies the similarities and differences in how LA and CLAU exert their effects. For example, it nicely shows how CLAU has a specific effect on intercalary leaflets, while CLAU and LA have an additive effect on primary, secondary and tertiary leaflets. Text to this effect was added to the manuscript.

Other criticisms should also be considered.

As the editor and one who doesn’t read every tomato leaf paper, I enjoyed it and found most of the figures stunning.

Much appreciated.

I was not as pleased with the transcriptome data as it is very hard to compare across different experiments. Ideally, the different genotypes would be grown at the same time and carefully harvested to make sure similar sampling was carried out. I gathered you took already published data. Comparing microarray data with RNAseq data is not great. And we have no idea what tissue was used, what stage, etc.

We added details as to the developmental stage and tissue that was used in the generation of the different data sets (lines 262-269). Most of the data analyzed if from our own previous work, with similar sampling. This is now indicated more clearly in the text. The public dataset we used from Ichihashi et al., 2014, is also now more clearly indicated. We added a comment about the limitations of comparing RNAseq to microarray data (lines 297-299).

Here are some additional points you need to address.
What is the BLS promoter? As mentioned already, the reader’s need reminding of the timing of different promoters.

We added an explanation (lines 161-162) , a citation and a cartoon to Figure 3.

In Figure 5, there is quantification for adding GA, but not for the other panels. Please add.

We added quantification for all the panels (new panels 5M and 5N) and for all the leaflet orders in new Supplemental Figures S6 and S7.

Figure S2B, D do not seem to correspond to the statement “This is in accordance with CLAU being expressed later than LA during the morphogenetic window, and with the effect of stage-specific expression of TKN2 (Figure S2B, D and (20,29,30,39,44)).”

The text was changed accordingly. We deleted this confusing statement. The expression timing of CLAU and LA within the developmental window has been addressed and clarified throughout the text (see, for example, lines 149-151; 178-180).

Please say what SlGA20ox-1 is.

Corrected (lines 226-228). Many thanks. This is the tomato (Solanum lycopersicum) homolog for the Arabidopsis GA20ox-1 – GA biosynthesis enzyme.

Please tell us about TCSv2 which shows up in Figure 6.

TCSv2 is a synthetic CK responsive promoter, here driving VENUS expression. We added the information to the text (lines 246-247).

I don’t see the data for this statement “morphogenesis genes were significantly enriched in morphogenetic genotypes clausa, la-6, and overexpression of miR319 or TKN2, while differentiation genes were significantly depleted in these genotypes” in figure 7. Does it require looking at the supplemental data? Looking at supplemental data sets means that no one sees the data. If there are important comparisons, they need to be more accessible.

We added some additional bioinformatic analyses and present more data and extensive explanations in new Figure 7 and its legend. The core of the data previously included in supplemental dataset 4 is now included in Figure 7.

Reviewer's comments

**Comments to the Authors:**

Reviewer #1: Tomato leaf, with the formation of multiple leaflets, is one of the major model of compound leaf morphogenesis and is used to understand how complex shapes form in plants. The authors addressed here the question of the relationship between different pathways mediated by transcription factors, LA, CLAU and TKN2 and hormones, mostly cytokinins and gibberellins during tomato leaf morphogenesis. There is already an extensive literature about this type of questions, and the general principle of the effects of these pathways (ie that they affect the balance between morphogenesis and differentiation and hence the possibility to elaborate complex shapes).
To analyse the relationship between LA, CLAU and TKN2, the authors analyse an impressive number of lines in which the activity of these gene is modified using different strategies (loss-of-function mutant, gain-of-function, ectopic expression from heterologous promoters, expression of repressor fusion proteins). The phenotypic characterisation of these lines mostly remains at the mature stage level and some of the conclusions are contradictory (see below). Then, the authors show that CLAU modifies the GA content and response. A reduced sensitivity of LA to CK is shown, extend observations previously made in Arabidopsis. Finally, trancrriptomics provides data about the overlap between LA, CLAU and TKN2 pathways and their effect on the expression of cohorts of genes involved in morphogenesis and differentiation.
The paper suffers from some major weaknesses (see below). It provides a refining of the interaction between different pathways, but as such this is not a major contribution to the field. Instead, some data such as the different temporal action of these different pathways are mentioned in the paper, but are not supported enough by the data.

Many thanks. Our work indeed builds on previous analyses by us and others, taking advantage of previously investigated pathways to further probe developmental questions. We added this explanation to the text (lines 333-335). Specifically, it is not completely clear how the balance between morphogenesis and differentiation is regulated. The compound tomato leaf is an excellent model to investigate the regulation of this balance, exactly because many regulatory factors and hormones have already been identified and data exists about them. We took advantage of these available systems and factors, and were able to determine how different regulators of the morphogenesis- differentiation balance, CLAU and LA, operate in mostly parallel genetic pathways- LA "opens" the developmental window for CLAU, and both their activities are affected by TKN2. CLAU and LA pathways are also "funneled" into the downstream ratio between CK and GA. A wide transcriptomic analysis of independent datasets confirms our molecular observations and suggests possible avenues for continued research into the morphogenesis-differentiation balance.

Major comments :

Tomato develop different types of leaflets (primary, secondary, interacallary) that form at different moments on the leaf primordium and at different relative positions. Such distinction between the different types of leaflets in not made here (eg Fig1MN)

We thank the reviewer for carefully reading and providing insightful suggestions to improve our manuscript. As detailed above in the response to the editors, in the revised version we added quantification of all leaflet orders to all the leaves in the different experiments, with the exception of Figure 4. This upgraded our analysis, provided us some more insights which are discussed in the revised text, and strengthened our results and conclusions regarding how exactly the three regulators act and affect similar but partially distinct aspects of leaf development, regulating differentiation and morphogenesis.

Also, the early stages of the leaf morphogenesis, that would give some hints about the dynamic process of leaf shape elaboration are not shown. It is therefore difficult to understand the developmental defects that lead to the perturbed mature leaf morphologies that are shown. Typically, la-2+/ clausa and FIL>>CLAU or clausa and FIL>MIR319 are described as having the same leaflet number (Fig 1MN), yet their morphologies are different. With the data provided, it is unclear if these lines indeed develop in a similar way or if they reach a similar leaflet number by two totally distinct trajectories. That going to the cellular level is important was shown by the same group in Shani et al., 2009, where they showed that expressing TKN2 or TKN2-SRDX under the FIL promoter leads to similar simple leaves which are however due to delayed or precocious maturation of the primordium.

This question was thoroughly addressed in previous work, which carefully analyzed early development of the mentioned genotypes. Specifically, up until the P4-P5 stage, leaves of the respective mutants are relatively similar (Kessler *et al.*, 2001; Ori *et al.*, 2007; Kang & Sinha, 2010; Burko *et al.*, 2013; Bar *et al.*, 2015, 2016). This is evident in revised supplemental Figures S5 and S8. Reducing LA and/or CLAU activity extends morphogenesis, leading to extensive leaf elaboration occurring in this extended period, typically after the leaf has unfurled. For example, in Bar et al., 2015, we showed that the number of leaflets in WT leaves plateaus around 50 days of plant age, while in *clausa*, it continues to steadily increase for another 20 days. Notably, total leaflet numbers in *clausa* were only increased about 20% when compared to WT in 6 week old plants, however, in 10 week old plants, the same *clausa* leaves followed throughout the experiments had an increase of more than 500% in total leaflet number when compared with the same WT leaves (Figure 5 in Bar et al., 2015). This is also evident from the fact that primary leaflet number is a relatively stable trait upon extended morphogenesis in *clausa* or *pFIL>>miR319* (Figure 1), as primary leaflets form first during leaf development (Kang & Sinha, 2010). The current manuscript builds on this analysis and goes a step further, aiming to understand how the activities of different effectors are coordinated to modulate leaf shape.

The leaflet order analysis we have added to the revised manuscript also addresses this question by providing additional insight into the dynamic process of leaf shape elaboration. This analysis indicated that CLAU is likely active along the leaf rachis, leading to a significant increase in intercalary leaflets upon its absence, while LA is not active in this domain. We have added discussion to this effect in the text (lines 123-129).

The interpretation of the role of TKN2 is not correct. For instance, it is mentioned in the abstract that “Maintaining a prolonged morphogenetic phase in early stages of compound-leaf development in tomato is dependent on delayed activity of several factors that promote differentiation, including … the morphogenesis promoting activity of the transcription factor TKN2 ». This conclusion is based on the effects of its overexpression, which does not necessarly mean that this is the role of the endogenous TKN2 gene.

The way observations following TKN2-SRDX expression are interpreted must also be more cautious. First, because, TKN2-SRDX overexpression does not fully mimic a TKN2 mutant. In other publications of the groups it is stated that TKN2-SRDX may down-regulate expression of TKN1 to 4. Second, because if promoters other than its own promoter are used, its expression pattern/level can be modified and the TKN2-SRDX fusion may repress targets that may not be the true TKN2 targets. Therefore, stating as in page 12 that “In addition, reducing TKN2 targets from the LA expression domain (LA>>TKN2-SRDX) resulted in similar phenotypes to those of La-2/+ mutants (Figure S2B), which stresses the important role of TKN2 downstream of LA. » is an overinterpretation.

Many thanks for this comment. The text was amended accordingly. Many previous works have indicated that TKN2 maintains a "morphogenetic" state. It is likely that the effects we observed with TKN2/ TKN2-SRDX stem primarily from is essential role in maintaining morphogenesis. We have added explanations and removed confusing test in several places to address this issue (see, for example, lines 153-154; 162-163; 168-171; 173-175; 178-180; 190-193).

Page 1, it is concluded that CLAU and LA act through parallel pathways. « These genetic analyses demonstrate that the effect of CLAU and LA on leaf development is partially additive, indicating that they promote differentiation in parallel pathways ». But later, it is concluded that LA defines the expression window of CLAU, which rather indicates an overlap between both pathways. And the authors also conclude that LA and CLAU converge on CK/GA. Therefore, the relationship between both pathways is not clear.

We have made amendments throughout the text to clarify.

According to our interpretation, 2 distinct questions can be addressed by the data. The first is the genetic pathway by which each of the effectors acts. The second is the developmental context in which they act. We think that the presented data suggests that LA and CLAU act in mostly parallel genetic pathways, but that LA, by acting earlier that CLAU in leaf development, affects the developmental context in which CLAU acts, and by that affects its expression window. The genetic analyses demonstrate that lack of LA exacerbates the effect of lack of CLAU, leading to super-elaboration, while CLAU overexpression rescues LA deficiency, and LA overexpression rescues CLAU deficiency. Hence, they act in likely mostly parallel pathways. This is also evident from the bioinformatics analyses, which we have expanded and elaborated upon in the new version of Figure 7. The incomplete rescue in some cases, and the differences in elaborated leaf forms, suggest that the pathways may not be 100% distinct. Regarding the developmental context, LA defines the expression window of CLAU spatially, since LA is active earlier in development and in a wider spatial domain. Increasing the activity of LA thus renders CLAU activity irrelevant, since the spatio-temporal domain in which CLAU is active is essentially abolished upon increases in LA activity. This is not a genetic overlap but an effect on the developmental dynamics. The two TFs converge on the balance between CK and GA via parallel pathways. Since the CK/GA balance in a downstream effector of final leaf form and elaboration, many TFs and pathways likely converge on this balance. In fact, this was one of the goals of our work- to use partially elucidated pathways to probe this convergence from different angles.

We conclude that CLAU and LA operate in mostly parallel genetic pathways, having different spatio-temporal expression patterns and likely different immediate downstream targets based on our transcriptomic analyses. Their activity "funnels" from different pathways into the CK-GA balance, which is a common downstream mechanism to regulate morphogenesis and differentiation.

We have added discussion to the text to clarify this (see, for example, lines 132; 149-151; 219-221; 324-330; 342-347; 391-394).

One reason for this could be that for the genetic analysis (Fig1), only partial loss-of-functions are used for LA and no real knock-out.

Respectfully, we are not sure we understand this comment *la-6* is a full LA knockout; overexpression of *miR319* further down regulates TCP3, 10 and 24.

Minor comments.

Page 11. « To better understand the genetic regulation of the balance between morphogenesis and differentiation, we examined the interaction between the TFs CLAU and LA » is very misleading as it suggests interaction between these proteins. Here the genetic interactions are analysed

The text was changed accordingly, with reference to the "genetic relationships" rather than "interaction" (lines 106-107).

Page 12. The title “Common TKN2-promoted pathways mediate extended morphogenesis » is not clear.

The text was changed accordingly, focusing more on the rolw of TKN2 in extended morphogenesis that occurs when CLAU and/or LA are down regulated (lines 153-154).

Page 12. Remind the reader of the expression pattern of the different promoters used, eg BLS.

The text was changed accordingly. Cartoons depicting the spatio-temporal expression domain of each promoter were added to Figures 1 and 3.

Page15. Indicate the tissue that was sampled for the RNAseq experiments

Added with thanks (lines 262-266).

Page 16. “DEGs confirm dependencies between the LA genotypes, with between about a third to half of the genes significantly upregulated in La-2/+ being significantly downregulated in la-6 and upon miR319 overexpression (Figure 7, Supplementary Data 1). » This is not shown in figure 7.

Figure 7 was amended extensively, with additional analyses added. The sentence highlighted by the reviewer referred to the Supplemental data set. This has been corrected in the text (lines 274; 276). We do not feel this result is an essential part of the manuscript, as it's more a quality control check for the microarray data. However, it can be added to Figure 7 if needed.

Some of the references used for TKN2 are not appropriate. The mentionned papers do not included this gene. Eg ref 40, 41, 43. In fact this gene has only poorly been characterised.

Corrected, thanks.

Page 20 : « Our results demonstrate a "gradient" of transcription factor activity…that is translated to leaflet number ». Which gradient of transcription factors are the authors talking about?

The text was changed. The inaccurate word "gradient" was removed. When comparing the phenotype of homozygous La-2 vs heterozygous La-2/+, CLAU overexpression, reducing CK (in CKX overexpression), and GA application can all substitute for the extra copy of La-2- such that a "dose" effect is observed (lines 111-113; 368-370).

Reviewer #2: The manuscript by Israeli et al. entitled “Coordinating the morphogenesis-differentiation balance by tweaking the cytokinin-gibberellin equilibrium” investigates the relationship between the several key transcription factors LA, CLAU and TKN2, and the plant hormones GA and CK, in the regulation of the morphogenesis-differentiation balance. Genetic analyses revealed that the expression levels of LA in different genetic backgrounds determined the expression domain and level of CLAU to affect the final leaf shape of tomato. The function of LA and CLAU is relied on the activity of target TKN2. The clausa mutant has an altered GA profile and reduced sensitivity to GA treatment, but LA reduces leaf margin sensitivity to CK. They also revealed that plant transcription factors and hormones GA and CK are coupled into tuning the final compound leaf shape.

The text is concisely written, and data are clear and solid. I have a few points to be addressed in order to improve the quality of this work.

（1） In the Figure 1J and 1M, they are probably from different stages of compound leaves. Please indicate the exact stages of each photo. For example, In the Fig 1J, the leaflet number of clausa mutant is less than that of Fig M.

Many thanks. All leaves depicted are of similar age, and the same leaf number, as indicated in the methods section. Leaf complexity is also affected by environmental factors, and can be variable from one experiment to the next. The experiments the results of which are presented in Figure 5 showed lower complexity than those the results of which are presented in Figure 1, for example. All experiments were comparative, with all controls included in each experiment separately. The leaf picture in Figure 1 which was identified by the reviewer as having a lower leaflet number than reflected from the quantification, was replaced with another, more complex leaf from the same experiment, to better reflect the quantitative results presented.

（2） In the Figure 7 legend ” Global expression analyses of LA, CLAU and TKN2 ” should being changed to “Global expression analyses of LA, CLAU and TKN2”.

Amended, thanks.

**Have all data underlying the figures and results presented in the manuscript been provided?**
Large-scale datasets should be made available via a public repository as described in the *PLOS Genetics* [data availability policy](http://journals.plos.org/plosgenetics/s/data-availability), and numerical data that underlies graphs or summary statistics should be provided in spreadsheet form as supporting information.

Reviewer #1: Yes

Reviewer #2: Yes

PLOS authors have the option to publish the peer review history of their article ([what does this mean?](https://journals.plos.org/plosgenetics/s/editorial-and-peer-review-process#loc-peer-review-history)). If published, this will include your full peer review and any attached files.


If you choose “no”, your identity will remain anonymous but your review may still be made public.


**Do you want your identity to be public for this peer review?** For information about this choice, including consent withdrawal, please see our [Privacy Policy](https://www.plos.org/privacy-policy).

Reviewer #1: No

Reviewer #2: **Yes:** Jianghua Chen
