# Supplementary material for: Coordinating the morphogenesis-differentiation balance by tweaking the cytokinin-gibberellin equilibrium
Source: PLoS Genet. 2021 Apr 26;17(4):e1009537. doi: 10.1371/journal.pgen.1009537 (PMC8102002; doi:10.1371/journal.pgen.1009537)
Supplement: Attachment — Submitted filename: 31-3-21 Decision letter - point by point.docx [file pgen.1009537.s018.docx]

Editor's comments:

The authors have done a great job addressing concerns of reviewers and myself.

Many thanks for a helpful and swift review process.

Please find a point-by-point response to the editor. All comments raised were addressed in the revised version of the manuscript.

There are a couple places where the figure is not correctly mentioned in the text, so please go through and check that carefully. For example, Figure 6C is mentioned incorrectly and there were perhaps a couple more.

We have carefully checked throughout the text and have made corrections to figure references.

The authors mention the reduction of TcS in La-2/+. Because there are no leaflets, there is definitely less of this reporter, but is there significantly less of the reporter in the margin that does exist?

Do you have a way to quantify the reduction?

Indeed. This comparison is difficult because *La-2* mutant leaf primordia are morphologically different and have less leaflets. We added quantification for all genotypes and developmental stages (new Figure S8), in which we quantified both mean fluorescence, representing the mean of the strength of the observed signal, and corrected total fluorescence, which calculates the integrated density of the fluorescence based on the size of the area being measured. Indeed, as expected, in WT leaf primordia, the mean *TCS:VENUS* signal decreases during development, as the CK signal decreases, while the corrected total fluorescence increases, because the area being measured is larger as the primordia grows. These two measurements, conducted on identically captured images from at least 5 plants from three separate experiments, show that there is a reduction in *La-2/+* in *TCS* driven expression in both mean fluorescence (from the P2 stage) and corrected total fluorescence (from the P3 stage). Overall, we observed no differences in the fluorescence level in the meristem of the different genotypes, and a reduction in *TCS* driven expression in *La-2/+* leaf primordia compared with WT. We added discussion of this to the text.

You bring up anthocyanin on page 28. That seems totally out of the blue and I am not sure it is needed. (Figure S9).

This is true. CK promotes, and GA inhibits, anthocyanin accumulation (Greenboim-Wainberg *et al.*, 2005; Fleishon *et al.*, 2011; Efroni *et al.*, 2013). We therefore measured anthocyanin levels in the different LA genotypes to show the effect of *LA* activity on CK sensitivity. We added a sentence to better explain the reason for this experiment.

**Efroni I, Han S-K, Kim HJ, Wu M-F, Steiner E, Birnbaum KD, Hong JC, Eshed Y, Wagner D**. **2013**. Regulation of Leaf Maturation by Chromatin-Mediated Modulation of Cytokinin Responses. *Developmental Cell* **24**: 438–445.

**Fleishon S, Shani E, Ori N, Weiss D**. **2011**. Negative reciprocal interactions between gibberellin and cytokinin in tomato. *The New phytologist* **190**: 609–17.

**Greenboim-Wainberg Y, Maymon I, Borochov R, Alvarez J, Olszewski N, Ori N, Eshed Y, Weiss D**. **2005**. Cross talk between gibberellin and cytokinin: the Arabidopsis GA response inhibitor SPINDLY plays a positive role in cytokinin signaling. *Plant Cell* **17**: 92–102.
